# The Role of Nucleophosmin 1 (*NPM1*) Mutation in the Diagnosis and Management of Myeloid Neoplasms

**DOI:** 10.3390/life12010109

**Published:** 2022-01-13

**Authors:** Katalin Kelemen

**Affiliations:** Department of Laboratory Medicine and Pathology, Mayo Clinic, Phoenix, AZ 85054, USA; Kelemen.katalin@mayo.edu

**Keywords:** nucleophosmin 1 (*NPM1*), mutation, myeloid neoplasm, acute myeloid leukemia, minimal residual disease (MRD)

## Abstract

Nucleophosmin (NPM1) is a multifunctional protein with both proliferative and growth-suppressive roles in the cell. In humans, *NPM1* is involved in tumorigenesis via chromosomal translocations, deletions, or mutation. Acute myeloid leukemia (AML) with mutated *NPM1*, a distinct diagnostic entity by the current WHO Classification of myeloid neoplasm, represents the most common diagnostic subtype in AML and is associated with a favorable prognosis. The persistence of *NPM1* mutation in AML at relapse makes this mutation an ideal target for minimal measurable disease (MRD) detection. The clinical implication of this is far-reaching because *NPM1*-mutated AML is currently classified as being of standard risk, with the best treatment strategy (transplantation versus chemotherapy) yet undefined. Myeloid neoplasms with *NPM1* mutations and <20% blasts are characterized by an aggressive clinical course and a rapid progression to AML. The pathological classification of these cases remains controversial. Future studies will determine whether *NPM1* gene mutation may be sufficient for diagnosing *NPM1*-mutated AML independent of the blast count. This review aims to summarize the role of *NPM1* in normal cells and in human cancer and discusses its current role in clinical management of AML and related myeloid neoplasms.

## 1. Introduction

Nucleophosmin (NPM1), also called B23 or numatrin, is a phosphoprotein expressed at high levels in the granular region of the nucleolus [1,2]. NPM1 is a multifunctional protein that is involved in many cellular activities that may be related to both proliferative and growth-suppressive roles in the cell. The human *NPM1* cDNA encoding a 294-amino acid protein was cloned in 1989 [3]. In mice, inactivation of the *NPM1* results in gene developmental defects and embryonic lethality at mid-gestation [4]. In humans, on the other hand, *NPM1* is involved in tumorigenesis. The *NPM1* gene can undergo a variety of genetic alterations, explaining its contributions to molecular pathogenesis of tumors of diverse histological origins. Genetic changes of the *NPM1* gene include chromosomal rearrangements and deletions in various hematological and solid tumors. The mutation of *NPM1* plays a unique role in the pathogenesis of acute myeloid leukemia (AML) and is seen in about 35% of AML patients [5], which makes *NPM1*-mutated AML the single largest unique group of AML. *NPM1* has a greatly heterogeneous role in the cell and interacts with both oncogenic and tumor-suppressing cellular functions.

## 2. NPM1 Protein in the Normal Cell

In the normal cell, NPM1, an abundant phosphoprotein of 37 kDa that is highly concentrated in the nucleolus, shuttles rapidly between the nucleus and the cytoplasm [6,7]. The shuttling activity and proper subcellular localization of NPM1 is crucially important for normal cell function. By shuttling between cellular compartments, NPM1 participates in a multitude of cellular processes, including transport of pre-ribosomal particles, ribosome biogenesis, response to stress stimuli such as UV radiation and hypoxia, regulation of chromatin condensation and decondensation, which has a crucial effect on DNA transcription and DNA repair, and interactions with tumor suppressors *p53* and *ARF*.

**Figure 1:**
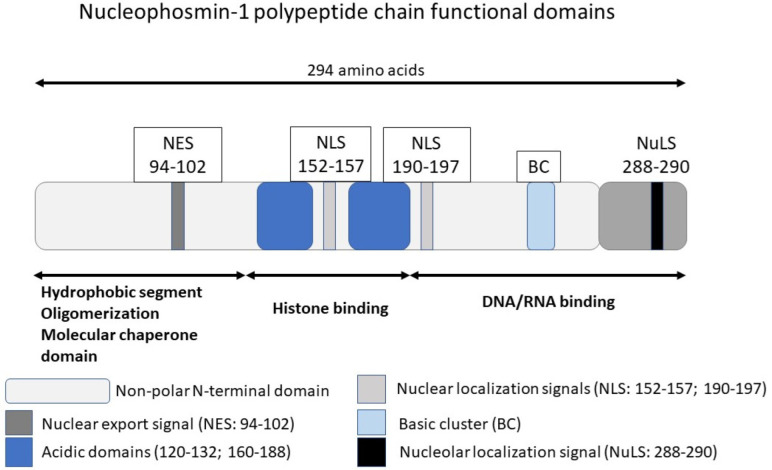
NPM1 polypeptide functional domains.

Within the structure of NPM1 protein, several functional domains account for various biochemical functions (Figure 1).

The N-terminal region carries a conserved non-polar sequence, with an important role as chaperone for proteins and nucleic acids [8,9,10]. In fact, all members of the family of nucleophosmins (Np) share this conserved region. This domain enables NPM1’s various functions, in addition to being a molecular chaperone. It can contribute to oligomerization, histone binding, nucleosome assembly, and acetylation-dependent transcription [11,12]. In addition to the N-terminal domain, different molecular domains are responsible for DNA/RNA binding functions. Various localization signals recognized within NPM1 include the nuclear export signal, the bipartite nuclear localization signal, and the nucleolar localization signal (Figure 1). The nucleolar localization signal (NuLS) is primarily responsible for anchoring NPM1 to the nucleolus, and it is dependent on the presence of two tryptophans at the C-terminus localized at W288 and W290, respectively [13,14]. Through these various domains, NPM1 can interact with many partners, such as nucleolar factors (nucleolin, fibrillarin), transcription factors (interferon regulatory factor 1 (IRF1), nuclear factor kappa B (NFkB)), histones (H3, H4, and H2B), proteins and enzymes (DNA polymerase-alpha), proteins of mitosis (NUMA), and proteins that respond to oncogenic stress (p53, ARF). NPM1 can associate with DNA [15] and RNA [10] and has endoribonuclease activity especially to ribosomal RNA (rRNA) [16]. The multitude of cellular activities NPM1 can interact with explains how NPM1 can exhibit both oncogenic and tumor-suppressor functions in the cell. Of the two existing alternatively spliced isoforms of NPM1 (B23.1 and B23.2), B23.1 is the more prevalent variant [17,18]. In the normal cell, NPM1 exists predominantly as an oligomer [19] and less commonly in the form of pentamers and decamers [20].

## 3. *NPM1* in Human Cancer

The role of *NPM1* in human cancer is remarkable for its involvement in tumors of diverse histological origins. At least three well-characterized genetic mechanisms can contribute: overexpression of the NPM1 protein, chromosomal translocations resulting in fusion products with *NPM1*, or mutation of the *NPM1* gene. In addition, *NPM1* maps to the 5q35 locus of chromosome 5, a region that is deleted in a subset of patients with de novo myelodysplastic syndromes (MDS). The partial or complete loss of chromosome 5 is also commonly observed in therapy-related MDS. The specific role of *NPM1* is yet to be explored in these subsets of MDS.

NPM protein is known to be overexpressed in various tumors, including carcinomas of gastric [21], colonic [22], ovarian [23], and prostate [24] origin. Overexpression of NPM1 mRNA has been shown to be an independent marker of recurrence and progression of bladder carcinoma [25].

On the other hand, chromosomal translocation and mutation of the *NPM1* gene are common genomic alterations in both lymphoid and myeloid hematologic neoplasms [26,27,28,29], with *NPM1* mutation being essentially unique to AML [5]. Chromosomal translocations may result in oncogenic fusion proteins, such as *NPM-ALK* in anaplastic large cell lymphoma, *NPM-RARα* (retinoic acid alpha) in acute promyelocytic leukemia (APL), and *NPM-MLF1* (myelodysplasia/myeloid leukemia factor 1) in MDS and AML. The mutant *NPM1* in AML leads to abnormal subcellular localization of the protein in the cytoplasm. A common feature in both mechanisms is the reduction in the total amount of NPM1 protein due to decrease in *NPM1* dosage, secondary to heterozygosity. The decreased dosage of *NPM1* could be potentially augmented if a negative-dominant effect of the mutated protein exists over the wild-type protein.

The different mechanisms described above do not necessarily identify whether *NPM1* will function as an oncogene, a tumor suppressor, or both in any specific scenario. The role of *NPM1* as a proto-oncogene is supported by the observation that cancer cells in the phase of growth and proliferation overexpress *NPM1*, and at the same time, apoptosis is inhibited. The level of NPM1 protein is higher in proliferating and cancerous cells than in quiescent cells [30]. This mechanism may include the effect of the *MYC* oncogene, since *MYC* is one of the regulators of *NPM1* transcription [31,32], or the stimulation of DNA polymerase-alpha activity by *NPM1* [33]. Although there are various mechanisms, evidence supports that the main role of *NPM1* in supporting cell growth relates to its role in the synthesis of ribosomes. The ability of NPM1 protein to bind nucleic acids [15,34], to act as a nuclear-cytoplasmic shuttle [6,7], and transport pre-ribosomal particles [35,36] designate NPM1 as one of the most important contributors in ribosome assembly. Thus, it is possible that the overexpression of NPM1 in cancer cells increases cell growth and proliferation primarily by providing ribosomes. Another important mechanism is related to inhibition of pro-apoptotic pathways by NPM1 through various indirect mechanisms, such as increased DNA repair, upregulation of proliferating cellular nuclear antigen (PCNA), which is an essential component of DNA repair activity, and downregulation of the tumor suppressor IRF1 [37]. These mechanisms were demonstrated in studies performed on NIH-3T3 fibroblasts that exhibited resistance to UV-induced apoptosis due to overexpressed NPM1 [37]. In summary, *NPM1* has various options for promoting tumor genesis and function as a proto-oncogene.

**Figure 2:**
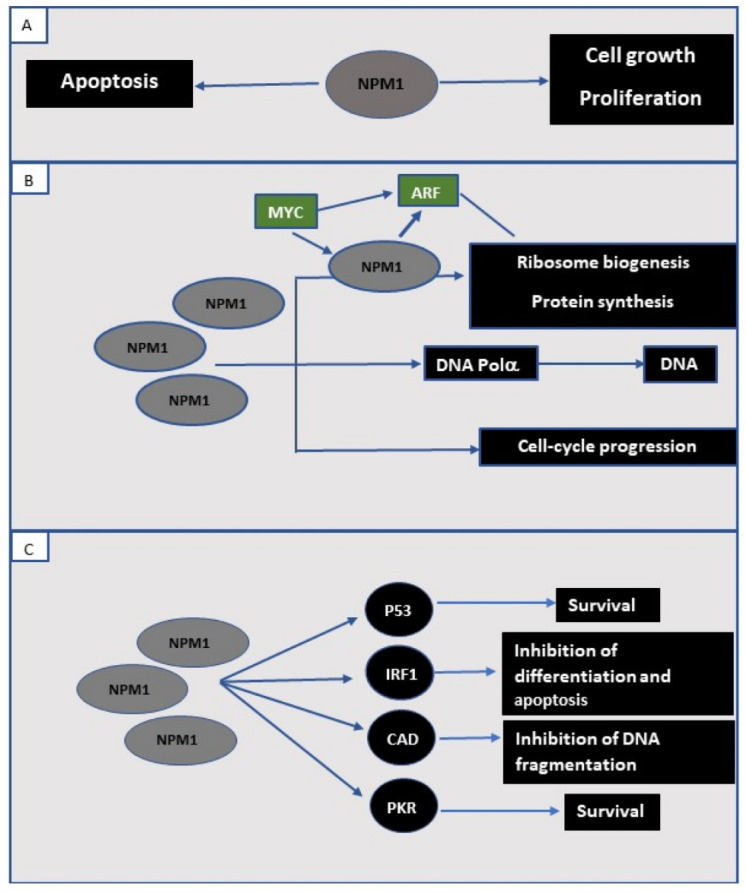
The effect of NPM1 overexpression in tumor cells. (**A**) In normal wild-type tissues, where a balance between cell proliferation and apoptosis is maintained, the expression of NPM1 protein is normal. In cancer cells, NPM1 is overexpressed and exhibits a proto-oncogenic effect by simultaneous stimulation of cell proliferation (**B**) and inhibition of apoptosis (**C**). A major contribution of NPM1 overexpression to cell growth and proliferation is the increasing ribosome biogenesis. NPM1 and several other ribosomal proteins respond to transcriptional regulation by MYC. Ribosome biogenesis is not the only mechanism since NPM1 also supports cell-cycle progression in addition to stimulating DNA polymerase alpha (DNA Polα). (**C**) Anti-apoptotic properties of NPM1 overexpression manifest through several different mechanisms. The stabilization of p53 and the downregulation of the catalytic function of eukaryotic initiation factor 2 kinase PKR support cell survival. The prevention of the transcription factor interferon regulatory factor 1 (IRF-1) from binding DNA inhibits apoptosis. Inhibition of caspase-activated DNAse (CAD) prevents DNA fragmentation and eliminates its pro-apoptotic activity.

Can *NPM1* function as a tumor-suppressor gene? NPM1 protein stabilizes ARF by binding to it inside the nucleolus and thus inhibiting its degradation [38,39]. The overexpression of exogeneous NPM1 has been shown to stabilize ARF [39]. On the other hand, ARF has been shown to inhibit nuclear to cytoplasmic shuttling of NPM1 [40]. These observations indicate that *NPM1* may be an important contributor to the ARF-mediated regulation of oncogenic stress, and as such, it could be considered as a tumor suppressor. Furthermore, increasing evidence suggests that during cellular stress, such as UV irradiation, NPM1 increases the stability of p53 by binding to MDM2 [41], a p53-specific ubiquitin ligase that mediates p53 decay [42]. The interaction between NPM1 and p53 may also take place downstream of p53—for example, by engaging GADD45a, a proapoptotic protein that is responsive to genotoxic stress. NPM1 directly binds to GADD45a and regulates its cellular localization [43]. Finally, NPM1 may have a role in the preservation of DNA stability via various molecular pathways, including the improving of the DNA repair process [44]. NPM1 is present in the centrosome and represents one of the substrates of CDK2-cyclin E complex [45]; thus, it contributes to the stabilization and regulation of mitosis. Cells of *NPM1* haploinsufficient mice (Npm+/−) show increased genomic instability and increased susceptibility to oncogenic transformation [4]. In summary, *NPM1* has a capability to act either as a tumor suppressor or a proto-oncogene, and its actual role may be determined by the balance between its various molecular partners and the level of *NPM1* expression (Figure 2).

### 3.1. Acute Myeloid Leukemia (AML) with Mutated NPM1; A Myeloid Neoplasm with Unique Features

The role of *NPM1* in human cancer has received a new spotlight with the discovery of mutations of exon 12 of *NPM1* in approximately 30% of adult de novo AML, and in 50–60% in those with a normal karyotype [46,47]. The latest updates of the World Health Organization (WHO) classification of myeloid neoplasm recognized AML with mutated *NPM1* as a distinct diagnostic entity [48].

While studying subcellular localization of NPM1 protein, Falini et al. observed a correlation between the presence of cytoplasmic NPM1 and certain clinical and biological features in AML [5]. In their study of 591 bone marrow samples of de novo AML, 35.2% of patients showed cytoplasmic NPM1, while cytoplasmic NPM1 was not observed either in 135 secondary AMLs or in 980 neoplasms other than AML. From a morphologic point of view, cytoplasmic NPM1 was observed in a variety of morphologic subtypes spanning from M0 to M7. More importantly, most cases with cytoplasmic NPM1 exhibited a normal karyotype and a good initial response to induction chemotherapy. These cases also showed an association with increased frequency of *FLT3* internal tandem duplication (*FLT3*-ITD) and lack of expression of CD34 and CD13 by flow cytometry [5].

It turns out that a novel mutation in exon 12 of the *NPM1* gene results in cytoplasmic localization of NPM1. Several different types of mutations exist. The most common type (called type A), representing about 70–80% of all mutations, features a four base pair nucleotide (TCTG) insertion at the position encoding the 288th amino acid residue, resulting in a frame shift of the downstream sequence [49]. As a consequence, the C-terminal amino acid sequence 286DLWQWWRKSL-COOH changes to 286DLCLAVEEVSLRK-COOH. After type A mutation, types B and D are the most frequent, representing 12% and 4% of all mutations in one study, while all other mutation types are less than 1% each, respectively [49] (Table 1).

Though numerous different sequence mutations exist in the *NPM1* gene (a total of 55 variants have been reported), all mutant proteins feature a critically important motif, notably the loss of at least one of W288 and W290, and they all share the last five amino acids [49]. Thus, despite the large number and variability of the mutations, all mutants invariably share a frame shift of the C-terminus. The frame shift directly relates to the cytoplasmic localization. Nakagawa et al. recognized that the frame shift results in a loss of nucleolar localization signal (WXW) and in a gain of nuclear export signal (LXXXVXXVXL) [50]. Falini et al. further confirmed that both of these signal changes are necessary for the relocation of the mutant NPM protein from the nucleus to the cytoplasm [51]. The correlation between the cytoplasmic localization of NPM1 protein and the mutation is strong. At the original discovery of *NPM1*-mutated AML, it was the cytoplasmic staining of NPM1 that directed attention to a subgroup of AML that turned out to become the single largest subtype of AML defined by a mutation [5]. The predominantly cytoplasmic staining suggests that there is an interaction between the mutant NPM1 and the wild-type product that results in retaining both products in the cytoplasm via heterodimerization [52]. The results of the German AML Cooperative Group supported the findings of previous studies: *NPM1* mutations were identified in 52.9% of AML cases [53]. Their report also confirmed the frequent association of *NPM1* mutations with *FLT3* mutations and additionally reported that *MLL* tandem duplication, *NRAS*, *KIT*, and *CEBPA* mutations were not common in *NPM1*-mutated AML [53]. There seems to be a bias in age distribution [49]. In AML patients younger than 35, *NPM1* mutations were less frequent than in those who were older than 35 years. Subsequent studies continued to add further layers of observations. Verhaak et al. reported that *NPM1*-mutated AML, while associated with normal karyotype and *FLT3* mutation is also associated with higher white blood cell counts [49]. In contrast with those patients with *NPM1* and *FLT3* mutations, *NPM1*-mutated AML patients without *FLT3*-ITD mutation represent a distinct subgroup of young adult patients with AML [54].

Of particular interest to diagnostic hematopathologists, multilineage dysplasia is observed quite frequently in bone marrow cells of AML with mutated *NPM1*, raising a differential diagnosis of AML with multilineage dysplasia [55]. It has become clear that in *NPM1*-mutated AML this type of dysplasia does not signify a worse prognosis and that the observation of dysplasia should not overwrite the diagnosis of AML with mutated *NPM1* [55,56]. Another observation, potentially important for disease follow-up, is a remarkable stability of *NPM1* mutations over time, reliably present at disease relapse and typically expressed across the entire leukemic population [14,46,57,58,59].

### 3.2. Prognosis of NPM1-Mutated AML

AML with mutated *NPM1* typically shows a good response to induction chemotherapy [5]. Cases with a normal karyotype in the absence of an *FLT3*-ITD mutation have a favorable prognosis [49,53,54,60,61,62]. In young patients with a normal karyotype and no *FLT3*-ITD mutation, the prognosis is comparable with that of patients with AML with t(8;21)(q22;q22.1) or AML with inv(16)(p13.1;q22) [54]. The presence of coexisting chromosomal abnormalities, observed in only 15% of patients, does not modify the prognostic effect of *NPM1* mutations [54,63,64]. Additional molecular mutations, if present, especially those of *FLT3, IDH1, IDH2*, and *DNMT3A* genes, may significantly alter the prognosis [46]. Though it is clear that the presence of *FLT3*-ITD mutations negatively influence the prognosis, it is to be pointed out that in younger adult patients, the prognosis is still better than in those patients who have AML with *FLT3*-ITD and wild-type *NPM1*, especially when the *FLT3*-ITD mutation is present at a low allelic ratio [65,66,67]. In older patients (aged older than 70 years), the negative prognostic impact of a coexisting *FLT3*-ITD mutation in *NPM1*-mutated AML is less clear [68]. The least favorable outcomes have been observed in cases with concomitant mutations of *NPM1*, *FLT3*-ITD, and *DNMT3A* [69].

## 4. Minimal Residual Disease (MRD) Monitoring in *NPM1*-Mutated AML

Unlike many other myeloid associated mutations, *NPM1* mutations are stable over time, usually documented at disease relapse and commonly expressed in the whole leukemic population [14,46,57,58,59]. Based on its homogeneous mutation pattern in AML, *NPM1* mutation may be considered an ideal leukemia-specific target for minimal residual disease/minimal measurable disease (MRD) detection [70]. Since the first RQ-PCR-based assay applied by Gorello et al. [71], RQ-PCR based assays have proven themselves as a reliable system for quantitatively assessing *NPM1*-mutated gene copies [71,72,73,74,75,76,77]. MRD assays most commonly include primer designed to the *NPM1* mutations type A, B, and D because these three mutations together represent the vast majority of AML cases (type A mutation represents 70–80% of all mutations, while types B and D together represent an additional 15–20%) [71,74,77]. The consensus from the European Leukemia Network (ELN) MRD Working Party recommends that AML patients with *NPM1* mutations undergo molecular MRD evaluation at the following clinical timepoints: at diagnosis, after 2 cycles of induction/consolidation chemotherapy, and at the end of treatment [78]. The selection of best sample—namely, bone marrow (BM) versus peripheral blood (PB)—remains controversial due to the observation that *NPM1*-mutated MRD may sometimes be undetectable in PB but that a BM sample shows detectable MRD at the end of treatment. Balsat et al. reported discordant results (a positive MRD in BM and negative result in PB) in 24.6% of cases [76]. Shayegi et al. reported differences between BM and PB MRD in 14.5% of cases [79]. If such a discrepancy is detected, *NPM1* MRD monitoring is recommended every 4 weeks for at least 3 months to evaluate for MRD increase [78]. Overall, the BM aspirate is generally accepted to be the best specimen for MRD evaluation [80].

*NPM1* mutation levels at diagnosis as measured by RQ-PCR did not have an impact on complete remission (CR) rate after induction chemotherapy, and other survival measures such as event-free survival (EFS) and overall survival (OS) were not influenced either [13,65,81,82]. Conversely, as it has been demonstrated recently by Patel et al., high *NPM1* variant allelic frequency (VAF) (>0.44) detected by next-generation sequencing (NGS) correlated with shortened median OS and shortened EFS. High *NPM1*-mutated allele burden at diagnosis by NGS was associated with unfavorable clinical outcomes [83]. This suggests that VAF determined by NGS may correlate better with a true clonal disease burden, which is different from the leukemic blast burden due to the multilineage presence of *NPM1* mutations in the BM.

In *NPM1*-mutated AML patients who previously reached an MRD negative status, serial molecular MRD evaluation may offer the benefit of predicting an impending relapse [80,84,85]. It has been documented by several studies that the reoccurrence of *NPM1* mutation may be documented as early as 2–3 months before a morphologic relapse becomes evident [73,74,79,84,86,87,88,89,90,91,92,93,94]. The median time from the detection of *NPM1*-mutated transcripts to the overt morphologic relapse was highly variable (median of 2.6 months, range 0.4 to 23.6 months) [91].

The role for allogeneic hematopoietic cell transplantation (allo-HSCT) in *NPM1*-mutated AML is still controversial. The prognostic value of pretransplant *NPM1*-mutated MRD has been evaluated in several studies [76,92,93,94,95,96,97]. Karas et al. demonstrated a negative prognostic impact of pre-transplant MRD positivity on EFS and OS in the patient population older than 63 [92]. In a study of Kayser et al., a significantly different OS was observed between pre-transplant MRD negative and MRD-positive cases (estimated 5-year OS 89% versus 40%, respectively). Other variables such as *FLT3*-ITD mutational status did not influence this difference [95]. Another interesting conclusion from these studies was related to the role of *NPM1*-mutated MRD for transplant selection. Notably, patients who achieved a >4-log *NPM1*-mutated MRD clearance did not benefit from allo-HSCT, but a survival benefit existed for those who failed to achieve clearance [76,80]. The clinical implication of these results is far-reaching, not only because *NPM1*-mutated AML represents the largest uniform subgroup within AML but also because these patients are classified as being of standard risk, for whom the best treatment strategy (transplantation versus chemotherapy) in unclear. It is now possible that the detection of persistent residual *NPM1*-mutated transcripts may provide additional guidelines for selecting the best treatment approach.

Finally, there may be a role for *NPM1*-mutated MRD monitoring in predicting relapse after allo-HSCT [73,79,88,94,97,98]. Several studies investigated the potential usefulness of *NPM1* mutation MRD detection in predicting relapse after allo-HCT. Based on the study of Zhou et al., highly sensitive NGS for *NPM1*-mutated transcript performed around day 28 after an allo-HSCT predicted a morphologic relapse in 15 of 18 cases (83%) [97]. Delsing Malmberg et al. used targeted deep sequencing for *NPM1* MRD at 3 months after allo-HSCT and observed that MRD positive result predicted relapse and was predictive of OS [94]. These early data indicate that patients with *NPM1*-mutated AML who received an allo-HSCT could benefit from *NPM1* MRD monitoring after transplant. Unfortunately, this benefit is decreased by the fact that preventive therapies, such as tapering if immune suppression, donor lymphocyte infusion, and HMA are not yet standardized [80,93,95,99].

Despite the many advantages of *NPM1*-based MRD monitoring in AML, various challenges exist. *NPM1* mutation is stable over the course of disease [57,100,101], and it has been detected at relapse even in patients who experienced more than one relapse, or relapses at extramedullary sites [57,100,101]. However, lately it became clear that in rare cases *NPM1* mutation may be lost at relapse [72]. The frequency of relapsed AML with undetectable *NPM1* mutations has been extremely variable, ranging from 1% [102] to 25% in small studies [103,104]. Cases that relapsed with undetectable *NPM1* mutations were believed to represent a new, therapy-related AML rather than a true relapse [57,72,91]. Later studies outlined the role of clonal evolution behind the phenomenon of lost *NPM1* mutation at relapse [72]. Kronke et al. compared paired BM or PB samples collected at diagnosis and at relapse and observed that *DNMT3A* mutations were present in both the primary and the relapse samples in every case with absent *NPM1* mutation at relapse [105]. This study strongly suggests the role of a common ancestral clone that acquired a *DNMT3A* mutation before acquiring an *NPM1* mutation, and the *NPM1* wild-type/*DNMT3A*-mutated clone gave rise to the relapsed AML [105]. In such cases, RQ-PCR-based *NPM1* MRD monitoring will result in negative results [104,105,106]. Another challenge to the RQ-PCR-based *NPM1* MRD monitoring is a potential switch of *NPM1* mutation subtype from D to A, a rare scenario that has been recently reported [107].

## 5. *NPM1* Mutations in Myeloid Neoplasms with <20% Blasts

Since the original discovery of cytoplasmic *NPM1* in de novo AML by Falini et al., a strong and specific association between *NPM1* mutations and AML has been observed [46]. *NPM1* mutations were not observed in any non-myeloid neoplasm. Though less frequent compared with de novo AML, *NPM1* mutations have also been observed in secondary AML (defined as progressing from either MDS or myelodysplastic/myeloproliferative neoplasm (MDS/MPN)) with a variable incidence ranging from 4.5% to 27% of cases [108,109,110,111,112,113]. Some of the studies were able to show that *NPM1* mutations were commonly detectable during the antecedent MDS phase, while in other cases, the MDS stage lacked the *NPM1* mutation [108]. In myeloid neoplasms other than AML, *NPM1* mutation has been detected in MDS (2%) and myelodysplastic/myeloproliferative neoplasms (MDS/MPN) (3%), with MDS/MPN represented by mainly chronic myelomonocytic leukemia (CMML). Of note, *NPM1*-mutated MDS most commonly represented MDS with excess blasts (MDS-EB) at diagnosis [114].

Several small retrospective studies reported that the rare cases of MN with *NPM1* mutation and <20% blasts has been characterized by a rapid progression to AML, usually within 12 months of diagnosis [46,109,115,116,117,118,119,120]. Early reports suggested that rapid progression of *NPM1*-mutated MN requires additional mutations, especially *FLT3* mutations [117]. Later, it became clear that MN with *NPM1* mutation and <20% blasts rapidly progress into AML even without additional *FLT3* mutations [119,120,121,122,123].

**Figure 3:**
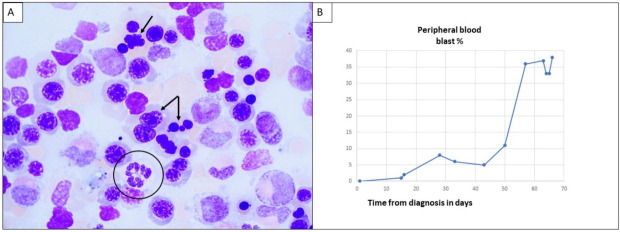
(**A**,**B**) A representative case of an *NPM1*-mutated myeloid neoplasm with less than 20% blasts.

Figure 3A shows the morphologic findings of the bone marrow aspirate of a 75-year-old man (Wright Giemsa, 1000 times magnification) who presented with macrocytic anemia (hemoglobin of 7.9 g/dL, MCV 105.3 fL) and thrombocytopenia (48 K/uL). The bone marrow shows dyserythropoiesis and dysgranulopoiesis without increased blasts. The arrows point to erythroid cells with dyserythropoiesis. The circle indicates a dysplastic neutrophil granulocyte (Figure 3A). A diagnosis of myelodysplastic syndrome with multilineage dysplasia was rendered. Routine karyotype analysis showed normal male karyotype. Next-generation sequencing showed *NPM1* mutation (VAT 45%) and *U2AF1* mutation (VAT 45%). Treatment was started with decitabine and venetoclax. Figure 3B. shows the change in peripheral blood blast percentage over the next two months after diagnosis. Sequential evaluation of the peripheral blood showed the occurrence of circulating blasts 15 days after the initial diagnosis, followed by a rapid increase in blasts percentage, reaching a diagnosis of AML by day 57 (Figure 3B).

A study of 144 CMML patients by Peng et al. described that *NPM1*-mutated CMML cases were characterized by more severe anemia, higher BM monocyte percentage, a more rapid transformation to AML, and decreased overall survival (OS) then non-*NPM1*-mutated cases, though this study did not reach statistical significance [119]. Another study by Vallapureddy et al. observed some of the same characteristics—notably, more severe anemia in *NPM1*-mutated CMML patients—and additionally described that *DNMT3A* and *FLT3* mutations were more common, while *TET2* and *ASXL1* mutations were less common than in CMML cases with no *NPM1* mutation [120]. This study also showed a higher risk of blastic transformation of *NPM1*-mutated CMML (63%) in comparison with *NPM1* wild-type CMML (18%) at a median of 5 months after initial diagnosis [122].

The largest multicenter cohort of MN with mutated *NPM1* and less than 20% blasts was reported by Patel et al. [123]. Myeloid neoplasms with *NPM1* mutations occurred in younger patients and were more commonly associated with a normal karyotype when compared with *NPM1* wild-type cases. The mutation landscape was also different, as *NPM1*-mutated MN more commonly showed *DNMT3A* and *PTPN11* mutations, while mutations of *ASXL1*, *RUNX1*, *TP53*, *IDH1*, *IDH2*, *FLT3*, *NRAS,* and *KRAS* mutations were less common than in the *NPM1* wild-type neoplasms [123]. When looking at the clinical treatment and outcomes, most patients with *NPM1*-mutated MN (73%) were treated with hypomethylating agents (HMA) upfront, and 39% progressed to AML at a median time of 5.2 months. As a contrast, none of the patients treated with intensive induction chemotherapy progressed to AML. These data suggest that a more intensive chemotherapy upfront may benefit patients with *NPM1*-mutated MN [123]. Montalban-Bravo et al. reported similar observations in a smaller patient cohort of 31 patients [121]. In their series, all cases showed multilineage dysplasia on morphologic examination, and as such, 61% of cases were designated as MDS. Overall, the *NPM1*-mutated MN were younger, had lower hemoglobin levels, and more commonly had a normal karyotype compared with *NPM1* wild-type cases. Interestingly, the clearance of mutation after therapy paralleled the disappearance of the dysplastic morphologic features. Complete remission (CR) rates were significantly higher after intensive chemotherapy than after HMA treatment [121]. Not every study shared this observation. Wu et al. reported a small cohort of MDS patients with *NPM1* mutation who achieved a favorable outcome after decitabine [124]. At present, a definitive conclusion for the best treatment of *NPM1*-mutated MN with less than 20% blasts cannot be drawn. The limited number of data available were derived from small retrospective studies, and they lacked a controlled clinical trial design [46,121,123]. Resolution of these controversial issues will require prospective multicenter clinical trials. Overall, due to the poor prognosis in most *NPM1*-mutated MN patients, HMA-based moderate intensity therapy could be considered inadequate. More intensive therapy could be considered for those *NPM1*-mutated MN patients who are fit, potentially including induction chemotherapy rather than MDS-directed treatment modalities [46,121,123].

An additional challenge relates to the pathological classification of these uncommon cases of MN showing *NPM1* gene mutation and less than 20% PB and BM blasts. The pathological diagnosis and subclassification has a direct impact on subsequent therapy. It has been long accepted that the presence of certain recurrent cytogenetic abnormalities including t(15;17)(q22;q12), t(8;21)(q22;q22), or inv(16)(p13.1q22)/t(16;16)(p13.1;q22) is enough to establish a diagnosis of AML even in the absence of 20% blasts [48]. In contrast, the same concept does not apply for the finding of *NPM1* gene mutation: myeloid neoplasms with less than 20% blasts do not meet current criteria for a diagnosis of *NPM1*-mutated AML. To further complicate the issue, multilineage dysplasia is a common finding in *NPM1*-mutated AML, yet the presence of *NPM1* mutation should have a priority over multilineage dysplasia as a disease-defining criterion [56]. The 2016 WHO classification has declared that *NPM1* mutation or biallelic mutation of *CEBPA* superseded multilineage dysplasia in the diagnostic classification. Based on presence of multilineage dysplasia, a case of *NPM1*-mutated MN with <20% blasts could be easily classified as MDS-EB or MDS/MPN. This diagnosis might sidetrack the patient for a subsequent diagnosis of AML with myelodysplasia-related changes (rather than AML with mutated *NPM1*) at the time when a transformation into AML occurred. Overall, AML with mutated *NPM1* may remain underdiagnosed. Forghieri et al. recommended an integrated molecular and immunohistochemical approach by demonstrating *NPM1* mutation in the bone marrow and extensive NPMc+ staining in more than 20% blasts in a bone marrow core biopsy to increase a successful diagnosis of AML with mutated *NPM1* [118]. Future studies will determine whether *NPM1* gene mutation may be sufficient to diagnose *NPM1*-mutated AML independent of the blast count [46,118]. The next revision of the WHO classification of myeloid neoplasm may provide definitive guidelines in this unresolved issue.

## 6. Summary

The availability of new molecular techniques, especially next-generation sequencing, introduced mutation testing into the routine diagnostic arena of myeloid neoplasms. Unfortunately, most of myeloid-associated mutations are not specific to any diagnosis, as they can be seen in individuals without disease and can be present in many different subgroups of disease (MDS, MDS/MPN, AML). Unlike most myeloid mutations, *NPM1* is remarkable for its specificity to a subtype of AML that is recognized as a specific diagnostic entity by the current WHO classification of myeloid neoplasms. In addition to its role in diagnostic subclassification, *NPM1* mutation determines a subgroup of AML with favorable prognosis, though this is influenced by the presence or absence of certain concomitant mutations, in particular *FLT3*-ITD and *DNMT3*. The *NPM1* mutation exhibits stability during persistent disease and relapse and has provided an opportunity for MRD testing in this subgroup in AML. Considering that *NPM1*-mutated AML represents the largest subgroup of AML and is classified as being of standard risk, the detection of persistent residual *NPM1*-mutated transcripts may provide additional guidelines for decision making between chemotherapy and hematopoietic cell transplantation. The best approach to the pathologic diagnosis and clinical management of myeloid neoplasms with *NPM1* mutation and less than 20% blasts is still unclear. These cases have been characterized with an aggressive clinical course and a rapid-progression AML, and they may benefit from aggressive treatment upfront. Future studies will determine whether *NPM1* gene mutation may be sufficient for diagnosing *NPM1*-mutated AML independent of the blast count.

## Figures and Tables

**Table 1:** Most common mutations of *NPM1* in AML.

Mutation	Sequence	Predicted Amino Acid
Wild type (*NPM 1.1*)	GAT CTC TGG CAG TGG AGG AAG TCT CTT **TAA** GAAAATAG	-DL**W**O**W**RKSL
Mutation A	GAT CTC TG**T** **CTG** GCA GTG GAG GAA GTC TCT TTA AGA AAA **TAG**	-D**L**CLA**V**EE**V**S**L**RK
Mutation B	GAT CTC TG**C** **ATG** GCA GTG GAG GAA GTC TCT TTA AGA AAA **TAG**	-D**L**CMA**V**EE**V**S**L**RK
Mutation D	GAT CTC TG**C** **CTG** GCA GTG GAG GAA GTC TCT TTA AGA AAA **TAG**	-D**L**CLA**V**EE**V**S**L**RK

The four inserted nucleotides are underlined. Stop codon is bold. The wild-type sequence features a nucleolar localization signal **W**X**W.** In the mutants, the nucleolar localization signal is replaced by **L**XXX**V**XX**V**X**L**, which represents a nuclear export signal, shown in the predicted amino acid column.
